# The Efficacy of Utilizing the Anatomage Table as a Supplementary Educational Resource in Osteology Instruction for First-Year Medical Students

**DOI:** 10.7759/cureus.46503

**Published:** 2023-10-04

**Authors:** Dharam Singh Rathia, Mrithunjay Rathore, Meryl John, Rahul K Ukey

**Affiliations:** 1 Anatomy, All India Institute of Medical Sciences, Raipur, IND; 2 Anatomy, Christian Medical College, Vellore, IND

**Keywords:** human anatomy, teaching-learning, virtual dissection table, osteology, anatomage

## Abstract

Introduction

Osteology is the detailed study of the structure of the bones. This study assesses the effectiveness of employing the 3D visualization tool Anatomage table as a learning adjunct to osteology training in first-year medical students by post-test evaluations related to the humerus, radius, and ulna bones.

Method

This study was conducted in first-year medical graduate students in the Department of Anatomy, All India Institute of Medical Science (AIIMS), Raipur, India. Students included in the study were divided into two groups by simple random sampling after voluntary consent. The study group students, Group A, were taught osteology by traditional teaching and visualizing bone with a tool, an Anatomage table. The control group (Group B) is for traditional teaching. The study involved demonstrating each group's humerus, radius, and ulna bones, with sessions lasting 60 minutes. After each topic, a post-test was administered. A total of 94 students for the test for the humerus bone, 98 students for the radius bones, and 85 students for the ulna bones responded to the post-test conducted after sessions. Descriptive statistics were assessed using mean and standard deviation. Independent sample t-tests compare the mean marks obtained post-test by two groups of students.

Results

The results indicated that students in Group A scored higher mean marks than their counterparts in Group B across all three bone post-tests, but the significance of the differences varied. For humerus, mean marks obtained by students of Group A (Anatomage table teaching) (mean±SD: 4.00± 1.10) were higher than those of Group B (traditional teaching) (mean±SD: 3.63± 1.36). Still, we do not observe a statistically significant difference in mean marks of students of Group A vs. students of Group B (P=0.166, P>0.05). For radius, we observe statistically higher mean marks among students of Group A (mean±SD: 3.72±0.944) compared to students of Group B (mean±SD: 3.22±1.08) (P=0.021, P<0.05). Similarly, for ulna, we observe higher mean marks for Group A (mean±SD: 3.18.00±1.55) as compared to Group B (mean±SD: 3.13±1.21) but do not observe a statistically significant difference in mean marks of students of Group A vs. students of Group B (P=0.875, P>.05).

Conclusion

Including the Anatomage table for visualization during osteology sessions yielded benefits for all three sessions. Future studies could employ more extensive and diverse samples to validate the findings further and incorporate qualitative methods to gain insights into students' perceptions of both teaching methods.

## Introduction

The skeleton and individual bones are typically used to explain the numerous landmarks and attachments in osteology lessons [1]. In small groups, students participate in sessions on human osteology, during which the instructor shows them various bony characteristics, how bones are kept and held in a standard anatomical position, and the importance of the directional terms used to refer to the human body [2]. Using textbook atlases, students can study human bones and related structures [3]. This method is referred to as a traditional one [4]. This teaching system has its reputation as the study suggests the importance of 3-D shape information extraction by the haptic system; that is, students learned anatomy using observational activities that included touch [5].

However, a few challenges experienced by the instructor during osteology sessions include difficulty taking each student through the minute features of a bone due to large class sizes and time pressure on the faculty and students [6]. Even with textbooks and atlas, instructors struggle to demonstrate linked muscles and associated vascular and neurological components [3]. Various studies suggest implementing supplementary visual aids as suitable resources for the study of osteology to increase student participation [4,7]. Before exposing students to cadavers, we can use 3D visualization tools during osteology sessions to show gross features, muscle attachment, and related structures [4].

3D visualization is the process of creating graphical content using 3D software. 3D rendering, excellent computer-generated imagery (CGI), and 3D graphics are similar [8]. Visualization is one of the vital tools for enhancing learning structures [4]. However, the effectiveness and efficiency of these tools need to be tested by comparing learning outcomes derived using visualization tools [9]. In addition to dry bones, osteology should be taught using visual aids using appropriate 3D visualization tools [10-13]. In a study on Turkish physiotherapy students, participant learning style is associated with significantly higher academic performance [14]. This tool could be helpful for visual learners as visual media help students visualize bony features, making the class more exciting and recall the facts [8,13]. Many researchers support that visualization helps enhance anatomy learning [1,4,12,13,15].

Vazquez et al. described the evolution of resources used in teaching anatomy. The resources include textbooks, anatomical atlas, wax models, DVDs, cadaveric dissections, prosected specimens, endoscopy, etc. [16]. With the advancement of technology, several multimedia techniques have served in teaching anatomy [16]. Rich et al. reported using online teaching modules to teach the structure of the bone [17]. The Anatomage Table (Anatomage, Inc., Santa Clara, California) is a platform for 3D real-human anatomy that is thoroughly segmented. Each organ's structures are precisely recreated in 3D [18]. Indian studies on the Anatomage table also found the students' perspective of learning gross anatomy using the traditional method and the Anatomage table [19-21]. Several studies have studied the utility of using the Anatomage table as an adjuvant tool in learning anatomy [21,22].

No specific studies have discussed the impact of the Anatomage table on learning osteology for undergraduates so far. The Anatomage table is manufacturer-specific. This project is non-funded and has no source of conflict by the manufacturer. This study aims to give an insight into using the Anatomage virtual dissection table in teaching osteology to undergraduate medical students and to assess the effectiveness of employing the Anatomage table as a learning adjunct to osteology training in first-year medical students.

## Materials and methods

The study was conducted in the Department of Anatomy, All India Institute of Medical Science (AIIMS), Raipur, India, after getting ethical clearance from the research cell and Institutional Ethical Committee AIIMS, Raipur, India (AIIMS/IEC/2023/1300, Date: 21.01.2023).

This study assesses the effectiveness of employing the Anatomage table as a learning adjunct to osteology training in first-year medical students. The first-year Bachelor of Medicine, Bachelor of Surgery (MBBS) students who had never been exposed to the osteology of humerus, radius, and ulna bones were included in the study after voluntary consent. By simple random sampling, more than 120 students were randomly divided into a study group, Group A, and a control group, Group B, for each osteology session.

Group A, teaching osteology using the Anatomage table: The study group students were taught osteology by visualizing dry bones, gross features, anatomical positions, muscle attachments, and other structures near the bones shown using the textbooks, atlas, and the Anatomage table. Using the home menu, the Anatomage table workflow includes the following: Gross anatomy - Male full body Asian - Visibility menu - Skeletal and muscular system - Upper limb bone - selected Humerus, Radius, and Ulna.

Group B, teaching osteology using a traditional method: The control group (Group B) is for traditional teaching. They were taught osteology by demonstrating dry bones, gross features, anatomical positions, muscle attachments, and anatomical structures near the bones shown using textbooks and an atlas without the Anatomage table. Three long bones of the upper limb region, humerus, radius, and ulna, were chosen to demonstrate osteology to both groups for 60 minutes.

For each topic, a post-test was conducted. Ten minutes were allocated for each test, with five multiple-choice questions on Google Forms. Questions were framed according to the osteology topic discussed. To reduce bias, demonstrators were kept blind for questions prepared based on topics. After the test, both groups were shown the Anatomage table so that no students were excluded from the learning process. A total of 94 students for the test for the humerus bone, 98 students for the radius bone, and 85 students for the ulna bone responded to the post-test conducted after class. Data from each test for the humerus, radius, and ulna were transferred from Google Forms, segregated using Microsoft Excel (Version 16.52), and analyzed using Jamovi software (Version 2.3). Descriptive statistics were assessed using mean ± SD. Independent sample t-tests compare the mean marks obtained from post-tests by two groups of students. A p-value of less than 0.05 was considered significant.

## Results

A total of 94 students, 32 (37%) (Group A) and 62 (63%) (Group B) for the test for humerus bone; 98 students, 39 (40%) (Group A) and 59 (60%) (Group B) for the radius; and 85 students, 33 (39%) (Group A) and 52 (61%) (Group B) students for the ulna responded the post-test conducted after class (Table 1).

**Table 1:** Table showing descriptive statistics for marks obtained in each test

Group Descriptives								
Post-test	Total number of students	Group	Number of students	Percentage of student	Mean marks	Median	SD	SE
Humerus	94	A	32	37%	4	4	1.1	0.183
		B	62	63%	3.63	4	1.36	0.172
Radius	98	A	39	40%	3.72	4	0.944	0.151
		B	59	60%	3.22	3	1.08	0.141
Ulna	85	A	33	39%	3.18	4	1.55	0.27
		B	52	61%	3.13	3	1.21	0.167

We tested and satisfied data for normality before computing mean and variance to compare the mean marks of two groups [23]. Marks obtained in the post-test for the three bones, across both groups, were normally distributed as reported by visual inspection of the Q-Q plot to test normality. There was no significant outlier, as evident by visual inspection of the box plot.

Before comparing the mean marks of two groups having unequal sample sizes, Levene's test of equality of variance assessed homogeneity of variance. Then, we performed a student t-test for homogenous, and for non-homogenous, we performed Welch's t-test to determine if there were differences in mean marks between Group A and Group B students [23].

Humerus demonstration

The variance was homogeneous as assessed by Levene's test of equality of variance (P=0.123, P>0.05). An independent sample t-test was run to determine if there were differences in mean marks between Group A and Group B students.

**Figure 1:**
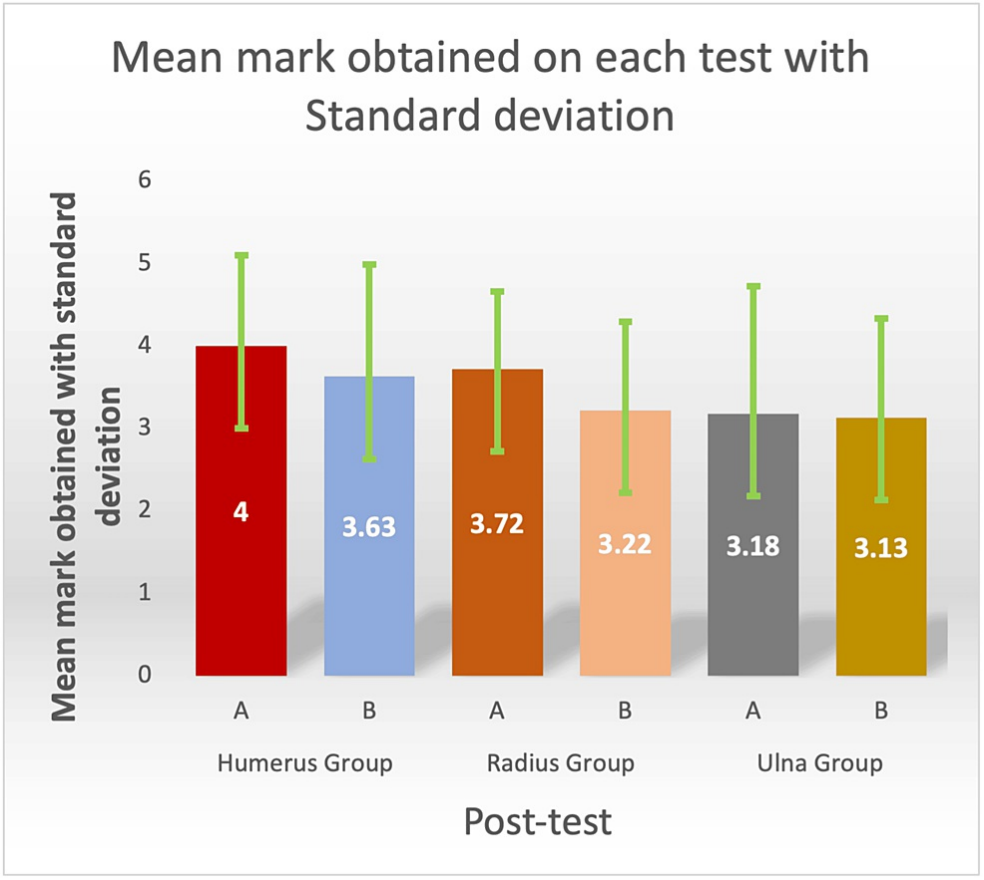
Mean mark obtained on each post-test with standard deviation

For humerus, mean marks obtained by students of Group A (Anatomage table teaching) (mean±SD: 4.00±1.10) were higher than those of Group B (traditional teaching) (mean±SD: 3.63±1.36) (Table 1, Figure 1).

Still, we do not observe a statistically significant difference in mean marks of students of Group A vs. students of Group B (P=0.166, P>0.05) (Table 2).

**Table 2:** Independent sample t-test for the comparison of the mean marks obtained in the post-test

Independent Samples T-Test							
						95% confidence interval	
	Statistic	df	p	Mean difference	SE difference	Lower	Upper
Humerus, Student's t-test	1.4	96	0.166	0.371	0.266	-0.157	0.899
Radius, Student's t-test	2.34	96	0.021	0.498	0.213	0.0753	0.92
Ulna, Welch's test	0.149	56.1	0.882	0.0472	0.317	-0.589	0.683

Interpretation for radius demonstration

An independent sample t-test was run to determine if there were differences in mean marks between Group A and Group B students. The variance was homogeneous as assessed by Levene's test of equality of variance (P=0.309, P>0.05).

For radius, mean marks obtained by students of Group A (Anatomage table teaching) (mean±SD: 3.72±0.944) were higher than group B (traditional teaching) (mean±SD: 3.22±1.08) (Table 1, Figure 1). We observe a statistically significant difference in mean marks of students of Group A vs. students of Group B (P=0.021, P<0.05) (Table 2).

Interpretation for ulna demonstration

There was no homogeneity of variance as assessed by Levene's test equality of variance (P=0.028, P<0.05). Thus, Welch's t-test was run to determine if there were differences in mean marks between Group A and Group B students [23].

For ulna, mean marks obtained by students of Group A (Anatomage table teaching) (mean±SD: 3.18.00±1.55) were higher than Group B (traditional teaching)(mean±SD: 3.13± 1.21) (Table 1, Figure 1). Still, we do not observe a statistically significant difference in mean marks of students of Group A vs. students of Group B (P=0.875, P>.05). We found that, for all three demonstration classes, the mean marks obtained by students of Group A (Anatomage table teaching) were higher than those of Group B students (traditional teaching) (Table 2).

## Discussion

To give an insight into using the visualization tool Anatomage, a virtual dissection table in teaching osteology, and to assess its effectiveness as a learning adjunct, we analyzed the students' performance in three bones post-test: humerus, radius, and ulna. The results indicated that students in Group A scored higher mean marks than their counterparts in Group B across all three bone post-tests, but the significance of the differences varied.

Significantly higher mean marks in the radius bone post-test suggest that the teaching method involving the Anatomage table positively impacted the students' understanding and retention of the radius bone anatomy. It indicates that the teaching method using visualization likely played a role in influencing the improved performance in Group A. These findings and suggestions are similar to studies on affect visualization in learning anatomy [4,12,24-26]. A higher mean mark but statistically insignificant post-test humerus and ulna suggest looking at other factors, such as individual variations in learning preferences and prior knowledge, study habits, and student motivation [14,27,28]. Maybe only visual learners benefit from visualization tools, and study with modified assessment methods is needed to test the learning of students [28]. Especially for the ulna, it might be due to factors such as ulna anatomy's complexity or other extraneous variables [27,29]. Apart from these results, our study has tried to overcome challenges during osteology sessions mentioned by Viswasom et al. instructors, and students have difficulty taking through the minute features of a bone due to large class sizes and time pressure [6].

Various interactive 3D digital models are available online and offline, and newer tools are being developed [1,7,10,24,28]. Through our study, we have tried to address one of the crucial questions raised by Erolin: "How can such digital models best be used to enhance student learning?" [1]. We used it as an adjunct to osteology and tested its utility. Our results suggest that visualization tools complement learning, such as textbooks and atlases, to overcome challenges during osteology sessions, as quoted by Raubenheimer et al. [3]. The study also addresses the issue raised in various articles: visualization tools will complement learning and not replace human specimens, especially real dry bones [1,10,17,22]. No such studies have mentioned the utility of the visualization of Anatomage in learning osteology. It is important to acknowledge the limitations of the study. Not all students responded to the post-test, which affected the sample size for each bone and might have affected the ability to detect differences. The study's findings suggest that adding the Anatomage table for visualization may offer advantages in enhancing bone anatomy learning outcomes.

## Conclusions

In conclusion, adding the Anatomage table for bone visualization during osteology sessions was beneficial for all three demonstration classes for humerus, radius, and ulna. Visualization tools should be employed to improve osteology learning. Even though many parts of the world have accepted virtual dissection tables, it is still a new educational modality in many countries. It needs to be further tested in various settings for utility and effectiveness. Future studies could use more extensive and diverse samples to validate the findings further and incorporate qualitative methods to gain insights into students' perceptions of both teaching methods.
